# Natural Product-Based Screening for Lead Compounds Targeting SARS CoV-2 M^pro^

**DOI:** 10.3390/ph16050767

**Published:** 2023-05-19

**Authors:** Jie Chen, Xiang Zhou, Lifeng Fu, Haiyu Xu

**Affiliations:** 1 Institute of Chinese Materia Medica, China Academy of Chinese Medical Sciences, Beijing 100700, China; 2 School of Chinese Medicine, Shenyang Pharmaceutical University, Shenyang 110016, China; 3 Key Laboratory for Research and Evaluation of Traditional Chinese Medicine, National Medical Products Administration, China Academy of Chinese Medical Sciences, Beijing 100700, China; 4 State Key Laboratory of Innovative Drug and Efficient Energy-Saving Pharmaceutical Equipment, Jiangxi University of Chinese Medicine, Nanchang 330004, China; 5 CAS Key Laboratory of Pathogenic Microbiology and Immunology, Institute of Microbiology, Chinese Academy of Sciences, Beijing 100101, China

**Keywords:** SARS CoV-2 M^pro^, natural products, high-throughput screening, lead compounds, in silico study

## Abstract

Drugs that cure COVID-19 have been marketed; however, this disease continues to ravage the world without becoming extinct, and thus, drug discoveries are still relevant. Since M^pro^ has known advantages as a drug target, such as the conserved nature of the active site and the absence of homologous proteins in the body, it receives the attention of many researchers. Meanwhile, the role of traditional Chinese medicine (TCM) in the control of epidemics in China has also led to a focus on natural products, with the hope of finding some promising lead molecules through screening. In this study, we selected a commercial library of 2526 natural products from plants, animals and microorganisms with known biological activity for drug discovery, which had previously been reported for compound screening of the SARS CoV-2 S protein, but had not been tested on M^pro^. This library contains compounds from a variety of Chinese herbs, including *Lonicerae Japonicae Flos*, *Forsythiae Fructus* and *Scutellariae Radix*, which are derived from traditional Chinese medicine prescriptions that have been shown to be effective against COVID-19. We used the conventional FRET method for the initial screening. After two rounds of selection, the remaining 86 compounds were divided into flavonoids, lipids, phenylpropanoids, phenols, quinones, alkaloids, terpenoids and steroids according to the skeleton structures, with inhibition rates greater than 70%. The top compounds in each group were selected to test the effective concentration ranges; the IC_50_ values were as follows: (−)–gallocatechin gallate (1.522 ± 0.126 μM), ginkgolic acid C15:1 (9.352 ± 0.531 μM), hematoxylin (1.025 ± 0.042 μM), fraxetin (2.486 ± 0.178 μM), wedelolactone (1.003 ± 0.238 μM), hydroxytyrosol acetate (3.850 ± 0.576 μM), vanitiolide (2.837 ± 0.225 μM), β,β–dimethylacrylalkannin (2.731 ± 0.308 μM), melanin (7.373 ± 0.368 μM) and cholesteryl sodium sulfate (2.741 ± 0.234μM). In the next step, we employed two biophysical techniques, SPR and nanoDSF, to obtain K_D_/K_obs_ values: hematoxylin (0.7 μM), (−)–gallocatechin gallate (126 μM), ginkgolic acid C15:1 (227 μM), wedelolactone (0.9770 μM), β,β–dimethylacrylalkannin (1.9004 μM,), cholesteryl sodium sulfate (7.5950 μM) and melanin (11.5667 μM), which allowed better assessments of the binding levels. Here, seven compounds were the winners. Then, molecular docking experiments were specially performed by AutoDock Vina to analyze the mode of interactions within M^pro^ and ligands. We finally formulated the present in silico study to predict pharmacokinetic parameters as well as drug-like properties, which is presumably the step that tells humans whether the compounds are drug-like or not. Moreover, hematoxylin, melanin, wedelolactone, β,β–dimethylacrylalkannin and cholesteryl sodium sulfate are in full compliance with the “Lipinski” principle and possess reasonable ADME/T properties, they have a greater potential of being lead compounds. The proposed five compounds are also the first to be found to have potential inhibitory effects on SARS CoV-2 M^pro^. We hope that the results in this manuscript may serve as benchmarks for the above potentials.

## 1. Introduction

Coronavirus Disease 2019 (COVID-19) is an infectious disease caused by SARS CoV-2 infection in organisms. The initial symptoms of the disease are similar to those of the “flu” and develop into life-threatening systemic inflammation and multi-organ dysfunction, such as acute respiratory distress syndrome (ARDS), pneumonia and renal failure. In severe cases, the virus can kill its hosts [1,2]. Controlling the viral disease has become a hot topic of interest for scientists as it has so far taken a significant toll on humanity, from its appearance in 2019 to its global spread and emergence in the public eye as a major pandemic. The SARS CoV-2 belongs to a new clade of β-coronavirus and has become the seventh coronavirus identified to infect humans. Among the first six diseases that cause symptoms of the common cold are 229E, OC43, NL63 and HKU1, while the zoonotic severe acute respiratory syndrome coronavirus (SARS CoV) and Middle East respiratory syndrome coronavirus (MERS CoV) are lethal [2]. As a positive-stranded RNA virus, coronavirus currently has the largest RNA viral genomes [3], and its genomes encode the expression of structural proteins including spike protein (S), envelope protein (E), surface membrane protein (M) and nucleocapsid protein (N), as well as the replicase protein ORF1ab (a polyprotein chain contains 16 non-structural, or functional, proteins) [4]. The main protease (M^pro^) is a part of the polyprotein chain, also named 3C-like protease, which cleaves firstly cleaves itself and then performs the same physiological function at 11 sites on the pp1ab chain, for a total of 14 sites there, subsequently producing functional protein fragments [5]. When it comes to the structure of the main protease, it is first of all a dimer and there are three structural domains per dimer, while the active site for physiological function resembles a pocket which is formed by numerous amino acid residues between structural domain 1 and 2. Slight variability in the amino acid sequence of the pocket over time has made it a focal point for drug targets [5,6]. Pfizer’s Paxlovid is the first oral small molecule drug targeting SARS CoV-2 M^pro^ approved by the US FDA in late 2021 [7]. The drug is composed of Nirmatrelvir, the main active ingredient in the treatment of the disease, and Ritonavir, a CYP3A4 inhibitor that is necessary to prevent the rapid metabolism of Nirmatrelvir by the body. This drug may have hepatotoxicity and drug–drug interactions [8]. In addition, as a peptide drug, it is easily hydrolyzed by amidase, which considerably affects its bioavailability. The Centers for Disease Control and Prevention (CDC) in America recently published an advisory about the potential for COVID-19 rebound after Paxlovid treatments. Studies have shown COVID-19 rebound to occur after Paxlovid [9], so the efficacy stability of Paxlovid needs to be considered. RdRp is a significant target for viral replication, in which Remdesivir and Molnupiravir are well-known drugs targeting SARS CoV-2 RdRp. Regrettably, the former has shown no significant benefits in clinical trials in China [10], and it probably has little or no effect on all-cause mortality from COVID-19 [11]; while the latter is limited in use due to genotoxicity [12]. The structural proteins of the SARS CoV-2 are primarily used to produce vaccines, and mostly related to the S protein [13]. Vaccines for several other structural proteins are rarely reported. Even though it has been reported, animals in experiments failed to avoid being infected by SARS CoV-2 virus [14]. Additionally, vaccines are primarily protective, not curative, so they cannot take the place of drugs.

**Figure 1:**
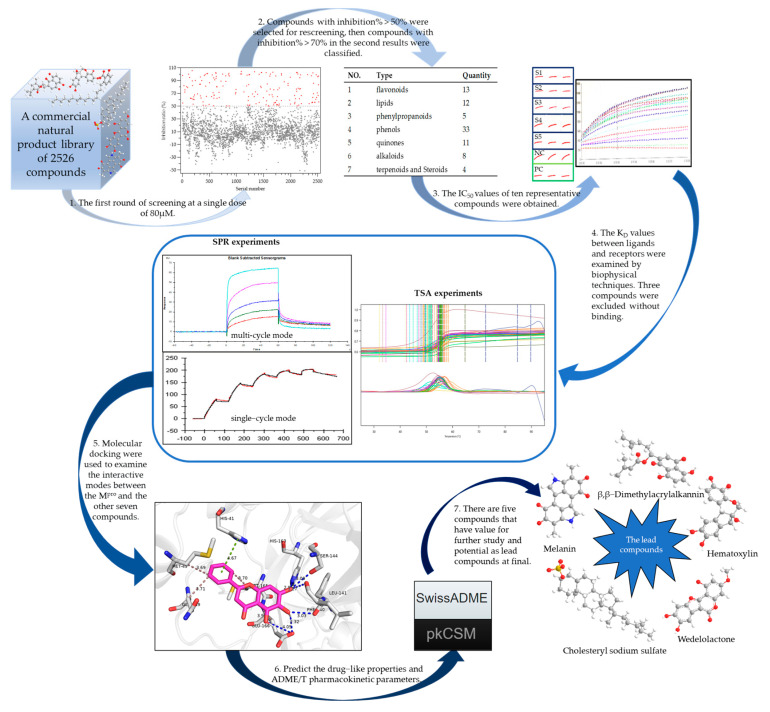
A graphical abstract of the experiments.

As the first surviving country in the COVID-19 outbreak, China has incorporated Chinese-style TCM into the treatment of patients, and several clinical trials demonstrating its clear clinical efficacy in treating COVID-19 [15,16,17,18,19]. The inclusion of “three TCM drugs and three herbal formulas” into the “Guidance for COVID-19: Prevention, Control, Diagnosis and Management” in China also indicates the significant role of TCM in the fight against COVID-19. However, the mechanism of action for herbal medicines is complex and the use of modern research tools to elucidate the mechanism of action at the molecular level is an effective tool. Numerous active ingredients in Chinese medicine are natural products, such as the globally recognized anti-cancer drug “Paclitaxel”, which is an active ingredient, can be found in the *Taxus chinensis (Pilger) Rehd* [20], and the anti-malaria drug “Artemisinin”, which is a sesquiterpene lactone extracted from the *Artemisiae Annuae Herba* [21]. The unique chemical structure complexity and bioactivity diversity of natural products have established the opportunity for the successful discovery of active lead compounds for the development of innovative drugs [22]. We hope that natural compounds with potential inhibitory effects on SARS CoV-2 M^pro^ can be found at the molecular level by high-throughput means, and we formulated the present in silico studies in order to determine whether they have the potential to serve as lead compounds [23] (Figure 1).

## 2. Results

### 2.1. Building the High-Throughput Screening Model

Building upon previous work foundations in our laboratory, we first purified SARS CoV-2 M^pro^ in preparation for the subsequent experiments, and the Fluorescence Resonance Energy Transfer method (FRET) [24] was chosen to test the protease activity as well as to screen potential compounds with an inhibitory effect. To ensure the reliability of our experimental results, baicalein, a natural compound previously reported to inhibit M^pro^ in vitro, was used as a positive control compound. Because the K_D_ value (Supplementary Figure S1) and the IC50 value (Supplementary Figure S2) measured in our experiments are consistent with those reported in the literature [25], this compound was also used to evaluate the feasibility of the screening system. After testing, we found that the gradient inhibition curves of baicalein measured at 200 μL and 100 μL of the experimental system practically matched (Supplementary Figure S2), indicating that the determination of fluorescence values was not affected even when the laser focus height at the liquid surface changed due to the different volumes, thus we chose the 100 μL system for the formal screening as it is more economical.

### 2.2. Screening the Prospective Natural Compounds

**Figure 2:**
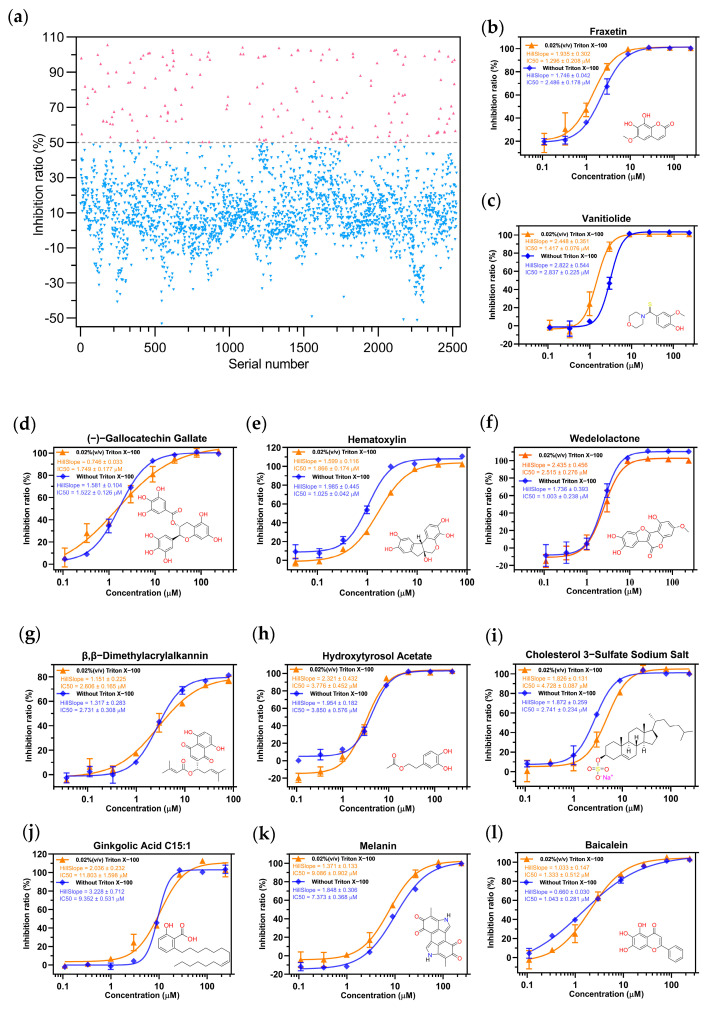
(**a**) Scatter diagram of inhibition rates’ distribution of 2526 compounds to M^pro^ at 80 μM. The gradient inhibition rates of compounds (**b**) fraxetin, (**c**) vanitiolide, (**d**) (−)–gallocatechin gallate, (**e**) hematoxylin, (**f**) wedelolactone, (**g**) β,β−dimethylacrylalkannin, (**h**) hydroxytyrosol ccetate, (**i**) Cholesterol 3–Sulfate Sodium Salt, (**j**) ginkgolic acid C15:1, (**k**) melanin, and (**l**) baicalein to M^pro^ under two conditions; error bars: mean ± S.D. of three independent replicates.

Prior to the experiments, we bought a commercial library of 2526 natural products (catalog number L6000, TargetMol, September 2020) from Shanghai Topscience Biochemical Technology Co., Ltd. for the inhibitor screening (Supplementary Table S1); it had been reported for compound screening of S protein [26,27], but not for M^pro^. Baicalein, a natural product known to have an inhibitory effect on SARS CoV-2 M^pro^ in vitro [25], was used as a positive control for the first round of screening of 2526 compounds at a single dose of 80 μΜ. The inhibition rates were calculated and the results are shown in Figure 2a. We selected compounds with inhibition rates greater than 50% for the next round of screening, and the minimum threshold of inhibition rate was raised to 70% for further selection of compounds. This resulted in 86 compounds that were further categorized into the following groups: (1) flavonoids (including flavonoids and catechins), (2) lipids (including fatty acids and phosphates), (3) phenylpropanoids, (4) phenols (including binary phenols and glycosides, ternary phenols, glycosides and salicylic acids), (5) quinones (including benzoquinones, naphthoquinones and anthraquinones), (6) alkaloids (including isoquinolines, pyrrolidines and morpholines), (7) terpenoids and steroids (Supplementary Table S2). Since lipids are essential nutrients for humans, providing energy and essential fatty acids to the body, and are also the building blocks of human cells and tissues, we excluded them from further experiments. The top-ranked compounds from the remaining six categories were selected for further testing to determine their suppression effect in a concentration range. These included (a) corylifol A, (b) (−)–gallocatechin gallate, (c) ginkgolic acid C15:1, (d) hematoxylin, (e) fraxetin, (f) wedelolactone, (g) hydroxytyrosol acetate, (h) vanitiolide, (i) β,β–dimethylacrylalkannin, (j) sanguinarine, (k) melanin and (l) cholesteryl sodium sulfate. Through the experimental test, the compound corylifol A was unable to generate a whole curve within the concentration range, which meant this compound became less effective when measured again. At the same time, the compound sanguinarine was found to have no inhibitory effect on M^pro^ when retested, thus these two compounds were terminated in the experiments here. The experimental data mapping results showed the IC_50_ value of the positive compound baicalein to be 1.043 ± 0.281 μM (Figure 2l), which is consistent with what had been reported in the literature (0.94 ± 0.20) [25]; the IC_50_ values of other compounds measured experimentally were also at the micromolar levels (Figure 2b–k). For the purpose of eliminating false positive effects which were made by the colloidal aggregation of compounds, a kind of surfactant Trition X-100 was added into the buffer (50 mM Tris-HCl, pH = 7.3, 1 mM EDTA) with a volume ratio of 0.02% [28,29]; this buffer was then used to carry out experiments as the last step; and finally, the gradient inhibition rates under two conditions are described (Figure 2b–l).

The experimental results from both parts demonstrate that the dose–response relationship of the compounds remained unchanged after the addition of the surfactant Triton-100. Furthermore, the IC50 values were found to be within the same order of magnitude. The findings indicated that these compounds inhibiting the activity of the protein were not colloidal aggregates, ruling out the possibility of such non-specific binding [5].

### 2.3. Biomolecular Interaction Experiments to Detect Binding

**Figure 3:**
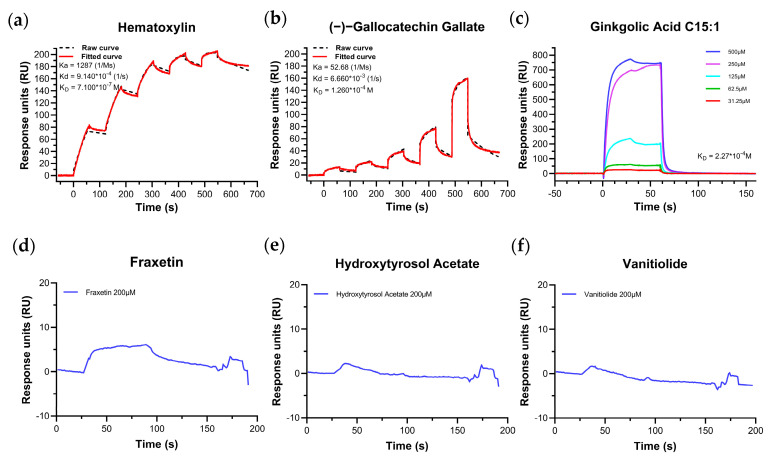
SPR-binding dissociation curves of compounds (**a**) hematoxylin (single−cycle), (**b**) (−)–gallocatechin gallate (single–cycle), (**c**) ginkgolic acid C15:1 (multi–cycle), (**d**) fraxetin, (**e**) hydroxytyrosol acetate, (**f**) vanitiolide. The gradient concentrations of the compounds are from low to high: 31.25 μM, 62.5 μM, 125 μM, 250 μM and 500 μM, and the single concentration was measured at 200 μM. The single-cycle kinetics were analyzed using 1:1 binding and the multi-cycle kinetics were analyzed by steady-state affinity.

For the above-screened compounds, we additionally employed the intermolecular binding techniques to verify whether there are bindings between them and M^pro^. Two biophysical means for estimating intermolecular binding affinity between M^pro^ and ligands were put into effect based on different experimental requirements. Among the numerous techniques, the Surface Plasma Resonance (SPR) technique is popular and widely used because it can respond to the binding affinity sensitively and reduce the false positive results of the experiment [30]. When the SPR technique was selected for the experiment, we took into account that the compounds were solubilized and stored in Dimethyl sulfoxide (DMSO), but the maximum tolerance of the instrument for DMSO was 10%, thus we selected six compounds that could be completely solubilized in a buffer (PBST, 7% DMSO) for this experiment. The solvent correction was necessary prior to the experiment, and the results showed that we had the correct compound concentration configuration, ruling out the interference of volume effects in the experiment. The SPR results showed that the compounds hematoxylin and (−)–gallocatechin gallate could bind to M^pro^ at different concentrations (Figure 3a,b), and the kinetic analysis showed that the two compounds bound to M^pro^ in a fast-binding and fast-dissociation mode, the affinity values were 0.71 μM and 126 μM, respectively. (−)–gallocatechin gallate had a K_D_ value that did not match the IC_50_ value, and its true binding potential should be in the hundreds of μM, indicating non-specific binding between the two during FRET experiments. There were no binding trends between M^pro^ and the compounds: fraxetin, hydroxytyrosol acetate and vanitiolide at 200 μM (Figure 3d–f), thus no multi-concentration measurements were made. The K_D_ value for the compound ginkgolic acid C15:1 in the multiplex mode was 227 μM (Figure 3c), but its binding-dissociation curve showed a clear non-specific binding trend.

**Figure 4:**
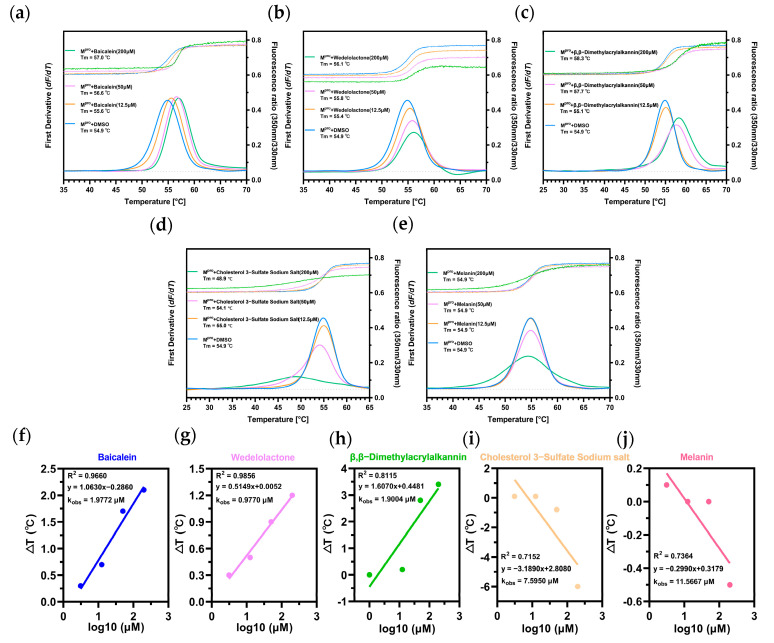
The denaturation profile and the curves of the first derivative of the fluorescence as a function of temperature (−dF/dT) of (**a**) M^pro^ + baicalein, (**b**) M^pro^ + wedelolactone, (**c**) M^pro^ + β,β–dimethylacrylalkannin, (**d**) M^pro^ + cholesteryl sodium sulfate and (**e**) M^pro^ + melanin, the T_m_ value is represented as the highest part of the curve. The K_obs_ for (**f**) baicalein, (**g**) wedelolactone, (**h**) β,β–dimethylacrylalkannin, (**i**) cholesteryl sodium sulfate and (**j**) melanin binding to M^pro^ were derived from plotting ΔT_m_ against log10 of compound concentrations and fitting to linear regression models.

Nano Differential Scanning Fluorimetry (nanoDSF) [31] technology was conducted in a Thermal Shift Assay (TSA) [32] to determine the changes in protein Tm values (∆Tm) after incubation with the baicalein and other compounds, which was used to assess whether binding between the small molecules and the protein occurred [33]. This is because when we performed the above-mentioned SPR experiments with these compounds, a little powder was precipitated by high-speed centrifugation after dissolving in buffer (PBST, 7% DMSO), which changed the concentration of the compounds and could potentially clog the column with sedimentation if SPR experiments were performed right. In TSA experiments, some compounds can increase the thermal stability of the protein after binding to the protein, making the melting temperature of the protein higher, and some compounds can decrease the stability of the protein after binding to it, with Tm values showing a tendency to decrease [33]. This technique is a simple, rapid and stable means of screening protein ligands, as the instruments used are disposable glass capillaries, and the liquid does not flow through the internal pathway of the instrument, causing no contamination or clogging of the instrument, nor does it require fixation of the protein sample. After the experiments, we can obtain the Tm values of proteins under different conditions (Supplementary Table S3). The first-order derivation of the temperature dependence of the fluorescence intensity was then plotted in the curve; there was a clear peak on the curve, and the temperature value corresponding to the peak was the denaturation temperature of the protein (Figure 3a–e). By linear regression of ∆T on the logarithm of the concentration, the intercept of the line on the x-axis corresponded to the concentration of Kobs; while not directly comparable, to some extent this can reflect the K_D_ value and is an excellent assessment of the protein–ligand stability [34]. By calculation, the Kobs values of compounds baicalein, wedelolactone, β,β–dimethylacrylalkannin, cholesteryl sodium sulfate and melanin were 1.9772 μM, 0.9770 μM, 1.9004 μM, 7.5950 μM and 11.566 μM (Figure 4), which were consistent with the experimentally determined IC_50_ values.

### 2.4. Molecular Docking Results

In order to understand the mechanism of protein–ligand binding, we carried molecular docking on research. Since the binding buffer used in our experiments was PBST (pH = 7.4), the selected compounds for docking were also required to be present at pH = 7.4. For this reason, we calculated the pKa values of each compound and found that hematoxylin, (−)–gallocatechin gallate, β,β–dimethylacrylalkannin and melanin existed as undissociated prototypes at pH = 7.4.

**Figure 5:**
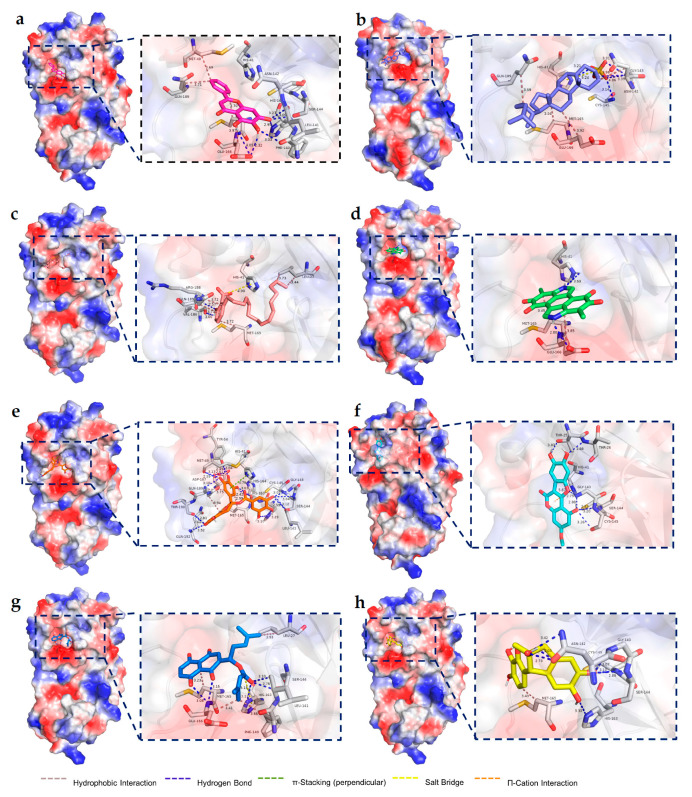
Binding pattern of compounds (**a**) baicalein, (**b**) cholesteryl sodium sulfate, (**c**) ginkgolic acid C15:1, (**d**) wedelolactone, (**e**) (−)–gallocatechin gallate, (**f**) melanin, (**g**) β,β–dimethylacrylalkannin, (**h**) hematoxylin to M^pro^ (PDB code: 7RFS) active site. The red and blue colors on the protein surface are the surface electrostatic potential, with red representing negative charge and blue representing positive charge.

The phenolic hydroxyl group on the benzene ring attached to the lactone ring in the structure of wedelolactone was ionized to make the oxygen atom negatively charged, and the phenolic hydroxyl group at position 7 on the phenyl chromogenic ketone ring of baicalein also existed in the form of the oxygen negative ion. The sulphate group in the cholesteryl sodium sulfate is linked to Na^+^ by ionic bonds, which become negatively charged sulphate bonds in water (Supplementary Figure S4). After identifying the molecular formulations of the compounds, we pretreated and docked the mol2 structure of the small molecules and the PDB structure of the protein M^pro^. The most stable conformation (the conformation with the largest absolute value of affinity) among the 20 docked structures was selected and the mode of interaction between the protein was analyzed (Figure 5).

Intermolecular interaction force analysis using pymol software and the PLIP website (Supplementary Table S4) showed that the phenolic hydroxyl group at positions 5/6/7 of the positive medicine baicalein chromogenic ketone formed multiple hydrogen bonds with the PHE140/LEU-141 main chains of M^pro^ and the side chains of GLU-166/SER-144/HIS-163, the chromogenic ketone ring had a hydrophobic interaction with the GLU-166 side chain; There is a π-π stacking between the side chain benzene ring and the side chain of HIS-41 and hydrophobic interactions with MET-49/MET165/GLN189, the crystal structure of the complex reported in the literature (PDB code 6M2N) [25] has S-π interactions and π-π interactions between the M^pro^ active site catalytic Cys145 and His41; these two residues constitute the significant catalytic dyad of SARS CoV-2 M^pro^ [5], and also NH2-π interactions between ASN-142 and baicalein. These three modes of interaction can also be generated in our docking results by determining the interatomic distances, only with an increase in interatomic distances compared to that of the co-crystalline structure, what is identical to the literature is the formation of hydrogen bonding interactions between the three phenolic hydroxyl groups of the chromogenic ketone ring and the surrounding amino acid residues, and the free phenyl ring forms numerous hydrophobic interactions with the surrounding amino acid residues, and the type of interaction forces present is consistent, which also indicates the reference value of our docking results.

We then molecularly docked the seven compounds obtained experimentally with M^pro^ to analyze the forces between the dominant conformers and the proteins, as well as their interaction types. Cholesteryl sodium sulfate is embedded in the substrate-binding pocket of the protein in long strips, with the head of the sulfate bond occupying the S1 subsite and mostly hydrogen bonding with the surrounding amino acids, and the tail of the hydrophobic chain occupying the S4 site and mostly hydrophobic interaction with the surrounding amino acids. Intermolecular hydrogen bonds were formed between the O atom of the sulphate ester bond in cholesteryl sodium sulfate and the main chain of GLY-143/CYS-145, and the side chain of HIS-41, which also created a salt bridge between the side chains of HIS-41, and the compounds also had hydrophobic interactions with the side chains of GLN-189/MET-165/GLU-166/ASN-142.

The carboxyl group of ginkgolic acid C15:1 extends into the S2 site to form a salt bridge between the imidazole ring of HIS-41, with hydrogen bonding between the phenolic hydroxyl group and the main chain of VAL-186/ARG-188, and the tail end of the hydrophobic chain is also present between LEU-27/MET-165/GLN-189 at the S1 site hydrophobic interactions. The two N’s of melanin form a hydrogen bond with the side chain of HIS-41/GLU-166 and there is a hydrophobic interaction between the ring and MET-165/GLU-166. The flavanol skeleton of (−)–gallocatechin gallate occupies the S4 site and the two benzenetriol moieties occupy the S1 and S2 sites, forming more interaction forces with the substrate-binding pocket of the protein. Multiple hydrogen bonds were formed between the phenolic hydroxyl groups on the two benzenetriol groups and the amino acid residues MET-49/LEU-141/GLY-143/SER-144/HIS-163/HIS-164/ASP-187/GLN-189 backbone, TYR-54 side chain, and the hydroxyl group at position 7 and the backbone of THR-190/GLN-192. There is π-π stacking between the substituted benzene ring at position 2 and the imidazole ring of HIS-41, which also has a hydrophobic interaction with the GLN-189/ MET-165 side chain, and between the flavanol skeleton and GLN-189. These forces nicely anchor the conformations of the compound. Hydrogen bonds were formed between the phenolic hydroxyl group of wedelolactone and the main chain of THR-25/THR-26/GLY-143/SER-144/CYS-145, between the O in the furan ring and GLY-143, and between the lactone ring and the nearby amino acid residue HIS-41 side chain imidazole ring, resulting in π-cation interactions. The side chain of β,β–dimethylacrylalkannin with an ester bond extends into the S1 site and the other side chain extends into the S1 site, with hydrogen bonding generated through the polar group. The carbonyl group of the naphthoquinone ring and the hydroxyl group on the adjacent benzene ring form hydrogen bonds with the main chain of GLU-166, the ester carbonyl group of the side chain forms hydrogen bonds with the main chain of LEU-141/SER-144, and the side chain of HIS-163, and there are hydrophobic interactions between the the methyl group at the end of the side chain and the side chain of LEU-27/PHE-140/MET-165, the hydrophobic interaction between the GLU-166 main chain, and the formation of a salt bridge between the carbonyl carbon of the ester bond with the HIS-163 side chain. The five- and six-membered rings of hematoxylin fold into two planes at 109.1° along a shared single bond, with the plane in which the five-membered ring is located reaching into the S1 site and the plane in which the six-membered ring is located facing the S2 site, with hydrogen bonding interactions between the catechol hydroxyl group attached to the five-membered ring and the side chain of amino acid residue HIS-163, the GLY-143/SER-144/CYS-145 main chain, there is also a hydrogen bonding interaction between the alcohol hydroxyl group attached to the pentacyclic ring and the side chain of ASN-142, and there is a hydrophobic interaction between the other catechol ring with MET-165.

### 2.5. Assessing the Druggability

Poor druggability is a major cause of late stage drug development failure, with up to 60% of development failures being due to poor ADME/T properties [35], so assessing the relevant properties before compound synthesis can effectively avoid wasting time and research funds. In order to ensure good oral absorption, the pre-drug needs to meet the “Lipinski principles” [36], which are widely used guidelines for drug-like properties, and good pharmacokinetics are also a prerequisite for further compound development. Predictions were made through the SwissADME [37] and pkCSM [38] websites and the results for important properties are listed below (Table 1).

Ginkgolic acid C15:1 did not fully comply with the “5” rules as its MLOGP was >4.15, while (−)–gallocatechin gallate had a hydrogen bond acceptor number >10 and a hydrogen bond donor number >5; it is considered to be in violation of the “Lipinski principle”, and if the compound is to conform to the rules, other groups need to be used to replace the excess hydroxyl group. The other five compounds are fully compliant and have good drug-like properties. In terms of compound absorption, the water-soluble drugs were better absorbed than the fat-soluble ones. The predicted results showed that ginkgolic acid C15:1 and cholesteryl sodium sulfate were poorly water soluble, while the rest of the compounds were amply water soluble.

Intestinal absorption could reach above 80% for all compounds except β,β–dimethylacrylalkannin and (−)–gallocatechin gallate, indicating that these compounds might have high oral bioavailability. The plasma protein binding of all these compounds was above 70%, indicating that the drug can be adequately transported in the blood, and the continuous release of the drug in the blood can maintain a stable blood concentration and exert a better therapeutic effect, and none of these compounds can cross the blood–brain barrier, so there is no concern about side effects on the brain. In terms of metabolism, the two main isoforms of CYP450 responsible for drug metabolism are 2D6 and 3A4. All compounds are not substrates of 2D6, and most are not substrates of 3A4, which prevents them from being metabolised quickly by the body.

The total clearance and bioavailability are related for compounds with high absorption and low clearance which can exert therapeutic effects in vivo for a longer period of time; ginkgolic acid C15:1 has a high total clearance compared to other compounds, while the high absorption and low metabolism nature of other compounds allows for a longer time to exert therapeutic effects in vivo. No significant cardiotoxicity, oral acute toxicity/chronic toxicity, hepatotoxicity or skin irritation were predicted for these drugs. Collectively, these compounds, particularly hematoxylin, melanin, wedelolactone, β,β–dimethylacrylalkannin and cholesteryl sodium sulfate, were predicted to be non-toxic, with reasonable pharmacokinetic (PK) properties, and have potential as lead compounds.

## 3. Discussion

Non-specific binding is present in all surface chemistry techniques and at all solid–liquid interfaces, it is also inevitable in SPR experiments. The kinetic profile of ginkgolic cid C15:1 showed a heterogeneous binding; it was a significant non-specific binding when the SPR experiment was performed, the slower phase of the binding curve never reaching equilibrium, and an incomplete dissociation then followed. However, there was neither a high response value nor a non-specific binding curve in the first channel through which the same buffer flowed as a control, ruling out the possibility that it was the components of the buffer causing the non-specific binding. Considering that the structural backbone of the compound consists of a hydrophobic benzene ring with a long chain alkane, and that the substituted group of carboxyl groups is ionized in the experimental buffer system and thus negatively charged, it is speculated that the binding curve is abnormal due to non-specific binding caused by a hydrophobic interaction and electrostatic adsorption.

The K_D_ value for (−)–gallocatechin gallate did not correspond to the IC_50_ value; this means the actual binding capacity of the compound to M^pro^ is weak, which is not difficult to understand. This is because the K_D_ value is calculated by measuring the association rate constant (kon) and the dissociation rate constant (koff), which are related to the concentrations between the two bound molecules and reflects the strength of the binding ability between them. Whereas the IC_50_ value is obtained from competitive biochemical experiments, where there are also substrates in the reaction system, inhibitors and substrates competing for the binding site of the protein, involving more variables and changing the concentration of any one of them will have an effect on the IC_50_. In addition to this, it had been reported in the literature that some compounds fluoresce on their own under laser excitation [39]; this will make the calculated result not the actual inhibition rate, so this possibility cannot be excluded. If the exclusion of the influence of the compound itself on the IC_50_ value is intended, one can use biophysical technology to determine the actual K_D_ value, or one can add a control group of compounds when performing enzyme activity experiments and then deduct them from the experimental results.

Small molecule inhibitors are a class of compounds with potential for drug discovery in the antiviral process that cannot be ignored. Baicalein [25], myricetin [40], shikonin [41] and some other components present in herbal medicine have previously been reported to have inhibitory effects on SARS CoV-2 M^pro^, and these natural product small molecules provide good examples for later studies. We have obtained a number of small molecules with potential as lead compounds through protein level experiments, and a compound search on the ETCM 2.0 [42] revealed that the final five compounds we screened are present in the plant *Caesalpinia sappan L*, *Poria cocos (Schw.) Wolf*, *Paeonia lactiflora Pall*, *Pinellia ternata (Thunb.) Breit*, *Wedelia chinensis (Osb.) Merr*, *Platycladus orientalis*(L.) *Franco and so on*. This may provide insight into the antiviral effects of Chinese medicine.

Although Paxlovid is already on the market, its “Paxlovid rebound” [9] has led to reservations about the stability of its efficacy, making the search for compounds with better stable effects on M^pro^ still relevant. In this study, advanced biophysical methods were used to determine the binding affinity between the compounds and M^pro^, and accurate K_D_ values were obtained, too. The affinity is in the same quantitative level as the inhibition effect, indicating the reliability of our experimental results. The five compounds screened in this manuscript are the first to be reported to have potential inhibitory effects on SARS CoV-2 M^pro^. However, the results are limited as the overall experiments only involved M^pro^. To further explore the antiviral effects of screening compounds, it is not only necessary to verify at the cellular level, but also necessary to conduct animal experiments to determine its efficacy in vivo. Single-component studies do not fully consider the synergistic effects of complex components in the body [43,44]. In a word, further experimental verification of the specific effects is required.

## 4. Materials and Methods

### 4.1. Materials

The Natural Product Library for HTS (catalog number L6000, 50 μL per hole, 10 mM, TargetMol, Shanghai, China, September 2020) was purchased from Shanghai Taosu Biochemical Technology Co., Ltd., including the following: fraxetin (catalog number T2909, TargetMol), vanitiolide (catalog number T0243, TargetMol), (−)–gallocatechin gallate (catalog number T3807, TargetMol), hematoxylin (catalog number T1686, TargetMol), wedelolactone (catalog number T3384, TargetMol), β,β–dimethylacrylalkannin (catalog number T5703, TargetMol), hydroxytyrosol acetate (catalog number T8168, TargetMol), cholesteryl sodium sulfate (catalog number T5239, TargetMol), ginkgolic acid C15:1 (catalog number T6S2123, TargetMol), melanin (catalog number HY-113485, MedChemExpress, Shanghai, China), baicalein (catalog number T2858, TargetMol), sanguinarine (catalog number T2781, TargetMol), corylifol A (catalog number T4S0145, TargetMol), M^pro^ fluorescent substrate (MCA-AVLQSGFR-Lys(Dnp)-Lys-NH2,C223JGC240-1/PE0977, GenScript, Nanjing, China), Sensor Chip SA (10291121, GE Healthcare, Shanghai, China), NHS-PEG12-Biotin (21313, Thermo Scientific, Beijing, China), ultrafiltration centrifuge tube (UFC901096, Millipore), 96-well Microtiter™ microplate (2305-11, Thermo Scientific), black 96-well plate (655077, Greiner Bio-One, Beijing, China), Triton™ X-100 (T109026, Aladdin, Beijing, China), Eppendorf tube (23-2052LK, Crystalgen, Beijing, China).

### 4.2. Methods

#### 4.2.1. Building the High-Throughput Screening Model

##### Protein Purification

The cells of *Escherichia coli* strain BL21(DE3) expressed in the recombinant protein were dissolved in lysis buffer (20 mM Tris, 50 mM NaCl, pH 8.0), sonicated for 30 min by an ultrasonic cell disrupter system (JY92-IIIN, Scientz Bio-Tech Co., Ningbo, China), the liquid was transferred to a centrifuge tube and centrifuged twice at 12,000 rpm for 20 min, and the precipitate was discarded. Proteins contain His tags and SUMO tags; the supernatants were flowed through the HisTrap HP 5 mL columns (17524801, GE Healthcare) to retain the protein on the column, then the proteins were de-gradient eluted using buffer (20 mM Tris, 50 mM NaCl, 300 mM imidazole, pH = 8.0) and collected by an AKTA purifier. Then, they were centrifuged and changed to a digestion buffer (20 mM Tris, 150 mM NaCl, 1mM EDTA, 1mM TCEP. HCl, pH = 7.8); per 30 mL of protein, 1 mL of SUMO protease was added (P2312M, Beyotime, Shanghai, China), centrifuged at 8000 rpm, and purified by Ni columns to remove the SUMO tags. Then, it was purified by size exclusion chromatography using a Hiload 16/600 Superdex 75 pg column (28989333, GE Healthcare), which was equilibrated with buffer (20 mM Tris-HCl, 150 mM NaCl, 1 mM dithiothreitol, 1 mM EDTA, pH = 7.8), and the measurement of protein concentration was obtained after concentration.

##### Protein–Ligand Binding of Baicalein to M^pro^ Supported by ITC

To see if the affinity between the positive drug baicalein and M^pro^ was consistent with that reported in the literature, ITC experiments were performed. The purified M^pro^ were changed into titration buffer (10% DMSO, 25 mM Tris, pH = 7.3) using the 75 pg chromatographic column, and the compounds were diluted in the buffer, with a final DMSO volume ratio of 10%, as well as keeping the dilution buffer exactly the same as the titration buffer. At 25 °C, protein concentrations ranged from 0.3 to 1 mM and compound concentrations ranged from 0.05 to 0.5 mM. Experiments were performed using an isothermal titration calorimeter (AFFINITY ITC LV, Waters, Beijing, China), titrating the compounds with protein at 2.5 μL per drop at 400 s intervals for a total of 20 drops; under the same conditions, the proteins were used to titrate the dilution buffer in order to decalculate the background heat. The results of control experiments were deducted and curve fittings were performed by NanoAnalyze software to calculate affinity values. We also used the heat generated by the titrating compounds with dilution buffer as references to assess the accuracy of the results.

##### Selection of FRET Systems

The initial system included the same volume of proteins and inhibitors in the 96-well plate, i.e., 80 μL each; the final concentration and volume of the fluorescent substrates were 20 μM and 40 μL, respectively. When the substrate is not cut, the energy generated by the fluorescence groups is absorbed and consumed by the adjacent quenched groups, making it impossible for the detector to detect the fluorescence. After M^pro^ cutting of the exclusive substrate MCA-AVLQ↓SGFR-Lys(Dnp)-Lys-NH2 [5,25,45], the fluorescence group(MCA-) was separated from the quenched group(Dnp-), meanwhile the fluorescence was released. The test system was set up without the small molecule inhibitors; however, the final volume per well was still 200 μL. With a fixed amount of substrate, the higher the concentration of protease, the stronger the enzymatic reaction capacity and the faster the initial reaction rate. Based on the previous work in the laboratory, a protease concentration with an initial reaction rate (or slope) of around 2000 was chosen for the construction of the complete system; the concentration range of M^pro^ was 5~30 μg/mL. Baicalein was used as a positive drug to verify if the system was successfully constructed. For economic reasons, the screening system was further optimized by reducing the overall volume to 100 μL, resulting in a final system of 100 μL = 20 μL fluorescent substrate + 40 μL protease + 40 μL buffer/inhibitor (Table 2). Taking into account the focus height of the excitation line and the depth of the wells of the 96-well plate, we compared the inhibition rates of baicalein under different conditions. The optimized system was not found to have a significant difference on the determination of fluorescence values, so the optimized system was used for the screening of compounds.

#### 4.2.2. High-Throughput Screening

The initial specification of the compound was a master mix (10 mM) with DMSO as the solvent to dissolve the powder, stored in 96-well plate. Given the poor water solubility of many natural products and the fact that most of them are small molecules, screening experiments were initially performed at a single high concentration of 200 μM. TE buffer (50 mM Tris-HCl, PH = 7.3, 1 mM EDTA) was chosen as the dilution buffer. Tris-HCl was selected to maintain pH to ensure the M^pro^ would not be denatured and lose its biological activity as an enzyme, while EDTA was added to bind some metal ions, and prevent some impurity proteases that require cofactors to function from causing non-specific binding. The procedure is as follows: add 2 μL of mother liquor to 98 μL of TE buffer and mix well, then pipette 40 μL of the compound solution to a black 96-well plate, add the same volume of protein solution and mix well and leave for 10 min at room temperature for co-incubation. The fluorescence intensity was measured using the CLARIOstar Plus multifunctional microplate assay system, with the excitation/emission light programmed to be 320 nm/405 nm, and the fluorescence was measured every 90 s for one cycle.

$$\mathrm{Inhibition}\;\%=\left[1-\frac{V0\left(\mathrm{sample}\right)}{V0\left(\mathrm{negative}\;\mathrm{control}\right)}\right]*100\%$$

The inhibition rate of the compound against M^pro^ at a final concentration of 80 μM was obtained in the previous step, and the concentration range in which the inhibitory effect of the compound exists needed to be further determined. For this purpose, we set multiple concentrations of the compound from almost mM to nM for the determination of the inhibition rate and used GraphPad software to perform a non-linear regression analysis of the compound in terms of concentration inhibition rate, calculating the half-inhibition rate of the compound and generating a quantitative effect relationship curve.

#### 4.2.3. Biomolecular Interaction Experiments

##### SPR Experiments

The biotinylation reagent was first configured as a 10 mM master batch. Since and the theoretical molecular mass of M^pro^ is 33796.8 Da [5], the amount of M^pro^ and biotinylation reagent was calculated in a 1:1 molar ratio using the following formula:

$$\mathrm{biotin}\;\left(\mu L\right)=\frac{\;c\left[M^{\mathrm{pro}}\right]\left(\frac{\mu g}{\mathrm{mL}}\right)}{\mathrm{MW}\left[M^{\mathrm{pro}}\right]\left(\mathrm{kDa}\right)}*V\left[M^{\mathrm{pro}}\right]\left(\mu L\right)*0.0001$$

The mixture was then incubated for half an hour at room temperature using a PBS buffer (pH = 7.40), and the unbound fraction was removed by column chromatography filtration. All SPR experiments were performed using a Biacore T-100 system with SA series chips. The chips were first activated with a 1 mM NaCl, 50 μM NaOH solution, followed by a 20 μg/mL protein solution flowing over the chip for fixation, and the injection stopped when the response value reached the maximum limit. The compound was dissolved to a concentration of 200 μM and the sample was injected to see if there was a tendency for the compound to bind at this concentration. The binding affinity value was determined at different concentrations in single-cycle or multi-cycle mode, and the binding affinity was determined by kinetic analysis or steady-state analysis.

##### Thermal Shift Assay

The compounds were diluted four times with DMSO in a gradient starting at 10 mM; each time, the concentration was a quarter of the previous. To be used, 1 mg/mL of protein was prepared, 49 μL of protein was obtained in an Eppendorf tube, 1 μL of compound solution was added and then mixed well by pipette; 49 μL of protein + 1μL of DMSO was used as the control group, using the nanoTemper N.T.48 equipped with a loading capillary and aspirating the sample until the capillary was filled, placing it in the order of the instrument slot, and testing whether the initial fluorescence value of the compound was greater than 2000; if yes, the temperature range was set from 25 °C to 95 °C, heating one degree more for each minute, and recording the various parameters during this process. At the end of the experiment, the data were exported for analysis and the denaturation curves were plotted using Graphpad 8.0 to check the denaturation temperature.

#### 4.2.4. Molecular Docking

The mol2 structural formulas of the compounds were downloaded from the ZINC database (https://zinc.docking.org/, accessed on 18 July 2022) and MarvinSketck (https://chemaxon.com/marvin, accessed on 20 July 2022) was used to calculate the pKa values of the compound to determine the molecular formulas of the compounds at pH = 7.4. The PDB file (7RFS) [7] for M^pro^ was downloaded from the PDB database (https://www.rcsb.org/, accessed on 30 June 2022). Pymol software (https://pymol.org/2/, accessed on 29 July 2022) was used to look at the structure of 7RFS, and through the location of the binding to the ligand, as well as the literature support [5], it was determined that its active cavity is in the crack between the one–two domain, similar in appearance to a “T-shape”. Then the compounds and proteins were pretreated using AutoDock Vina software (https://vina.scripps.edu/, accessed on 29 July 2022). The compounds were checked for rotatable bonds and saved in PDBQT format; the proteins were also saved in PDBQT format with BOX settings of center_x = 12.37, center_y = 0.371, center_z = 21.369, size _x = 26.25, size_y = 26.25 and size_z = 26.25 (this is the data after the test). The center of the grid box is just at the binding site. It is appropriate to adjust the number of points in the x/y/z dimension to fully include the active site, and the box should not be too large. The data were saved as a config file and the docked program 20 times. The most dominant conformation was exported as a PDB file, the interaction was analyzed using the PLIP website (https://plip-tool.biotec.tu-dresden.de/plip-web/plip/index, accessed on 30 July 2022) and the binding pattern to the protein was viewed and plotted using Pymol software.

#### 4.2.5. Assessing the Druggability

Using the SwissADME website (http://swissadme.ch/, accessed on 5 August 2022) to calculate the relevant physicochemical properties of the compounds and to see if the compounds met the five principles, the pkCSM website (https://biosig.lab.uq.edu.au/pkcsm/prediction, accessed on 5 August 2022) was used to predict the ADME/T properties of the compounds and assess whether the relevant and important pharmacokinetic properties of the compounds were reasonable.

#### 4.2.6. Ingredients-Herbal Counterpart

Click on the ingredients module in the ETCM v2.0 (http://www.tcmip.cn/ETCM2/front/#/, accessed on 18 April 2023) and then type the name of the compound in the search box; obtain “Herbs” at the bottom of the page.

## 5. Conclusions

In conclusion, natural products have been proven to be a promising source of lead compounds. Through the high-throughput screening of the SARS CoV-2 Mpro natural compound library, we have identified six valuable natural product classes and selected seven representative components using competitive enzyme activity inhibition assays and biomolecular interaction binding assays. These compounds are cholesteryl sodium sulfate (steroid), ginkgolic acid C15:1 (phenol), wedelolactone (phenylpropanoid), (−)–gallocatechin gallate (flavonoid), melanin (alkaloid), β,β–dimethylacrylalkannin (quinone) and hematoxylin (phenol).

The molecular docking results reveal that these compounds interact predominantly through hydrophobic interactions and hydrogen bonds with M^pro^. Furthermore, hematoxylin, melanin, wedelolactone, β,β–dimethylacrylalkannin and cholesteryl sodium sulfate conform to the “Lipinski principle” of drug likeness, and have reasonable pharmacokinetic properties, making them potential lead compounds for M^pro^ inhibitors. The inhibitory effects of these five compounds on SARS CoV-2 M^pro^ have been discovered for the first time. The experimental results in this manuscript provide a valuable reference for the development of lead compounds targeting viral proteases.

## Figures and Tables

**Table 1:** The drug-like properties and pharmacokinetic parameters of screening compounds.

Parameters	Hematoxylin	Melanin	Wedelolactone	Ginkgolic Acid C15:1	β,β–Dimethylacrylalkannin	Cholesteryl Sodium Sulfate	(−)–Gallocatechin Gallate
Molecular weight	302.282	318.288	314.249	346.511	370.401	488.71	458.375
LogP	1.3205	1.32664	2.8178	6.5001	3.6375	3.8772	2.2332
Rotatable bonds	0	0	1	14	5	7	3
Acceptors	6	4	7	2	6	4	11
Donors	5	2	3	2	2	0	8
Lipinski	Yes; 0 violation	Yes; 0 violation	Yes; 0 violation	Yes; 1 violation	Yes; 0 violation	Yes; 0 violation	No; 2 violations
Water solubility (log mol/L)	−3.424	−3.317	−3.393	−5.421	−3.583	−4.189	−2.933
Intestinal absorption (human) (% Absorbed)	84.974	99.755	95.067	88.537	63.844	100	46.991
Fraction bound (%) (human)	91.4	87.4	97.5	92.5	79.6	93.8	76.1
BBB permeability (log BB)	−1.135	−0.078	−1.262	−0.403	−0.076	−0.437	−2.058
CYP2D6 substrate	No	No	No	No	No	No	No
CYP3A4 substrate	No	Yes	No	Yes	No	Yes	No
CYP1A2 inhibitor	No	Yes	Yes	No	No	No	Yes
CYP2C19 inhibitor	No	No	No	No	No	No	No
CYP2C9 inhibitor	No	No	Yes	No	No	No	No
CYP2D6 inhibitor	No	No	No	No	No	No	No
CYP3A4 inhibitor	No	No	Yes	No	No	No	No
Total clearance (log mL/min/kg)	0.046	0.521	0.677	1.59	0.402	0.685	0.431
Max. tolerated dose (human)(log mg/kg/day)	0.403	0.485	0.751	0.066	−0.246	−0.75	0.605
hERG I inhibitor	No	No	No	No	No	No	No
Oral rat acute toxicity (ld50) (mol/kg)	2.07	2.457	2.363	2.111	1.819	2.359	2.764
Oral rat Chronic toxicity (LOAEL) (log mg/kg_bw/day)	3.032	1.963	1.377	2.643	1.954	1.141	3.844
Hepatotoxicity	No	No	No	No	No	No	No
Skin sensitization	No	No	No	No	No	No	No

**Table 2:** Compounds screening systems.

Group	Protein (μL)	Sample (μL)	Substrate (μL)	Final Volume (μL)
Sample group	40	40 sample	20	100
Positive control	40	40 baicalein	20	100
Negative control	40	40 buffer	20	100

## Data Availability

Data is contained within the article and supplementary material.

## Supplementary Materials

The following supporting information can be downloaded at: https://www.mdpi.com/article/10.3390/ph16050767/s1, Figure S1: The isothermal titration calorimetry result of M^pro^ to baicalein; Figure S2: The IC_50_ value and dose-effect relationship curve of baicalein at 100 μL/200 μL; Figure S3: Steady analysis fitting curves for ginkgolic acid C15:1 to M^pro^ and related parameters; Figure S4: The pKa value of each group of the compounds baicalein, cholesteryl sodium sulfate, ginkgolic acid C15:1, wedelolactone, (−)–gallocatechin gallate, melanin, β,β−dimethylacrylalkannin, hematoxylin; Table S1: Detailed information on 2526 natural products; Table S2: The 86 compounds obtained from the repeat sieve and their inhibition rates; Table S3: The Tm values for M^pro^ in the presence of different compounds; Table S4: Types of valence bonds between compounds baicalein, cholesteryl sodium sulfate, ginkgolic acid C15:1, wedelolactone, (−)–gallocatechin gallate, melanin, β,β−dimethylacrylalkannin, hematoxylin and M^pro^, and bonding positions.
