# Supplementary material for: Natural Product-Based Screening for Lead Compounds Targeting SARS CoV-2 Mpro
Source: Pharmaceuticals (Basel). 2023 May 19;16(5):767. doi: 10.3390/ph16050767 (PMC10222270; doi:10.3390/ph16050767)
Supplement: Supplementary file 1 [file pharmaceuticals-16-00767-s001.zip › 2.MDPI-Supplementary information.pdf]

Supplementary Materials

Natural Product-Based Screening for Lead Compounds Targeting SARS CoV-2 M^{pro}

Jie Chen [1,2], Xiang Zhou [3,4,*], Lifeng Fu [5,*] and Haiyu Xu [1,3,*]

[1] Institute of Chinese Materia Medica, China Academy of Chinese Medical Sciences, Beijing 100700, China
[2] School of Chinese Medicine, Shenyang Pharmaceutical University, Shenyang 110016, China
[3] Key Laboratory for Research and Evaluation of Traditional Chinese Medicine, National Medical Products Administration, China Academy of Chinese Medical Sciences, Beijing 100700, China
[4] State Key Laboratory of Innovative Drug and Efficient Energy-Saving Pharmaceutical Equipment,
Jiangxi University of Chinese Medicine, Nanchang 330004, China
[5] CAS Key Laboratory of Pathogenic Microbiology and Immunology, Institute of Microbiology,
Chinese Academy of Sciences, Beijing 100101, China
* Correspondence: flychow2001@163.com (X.Z.); fulf@im.ac.cn (L.F.); hyxu@icmm.ac.cn (H.X.)

This Word file includes: **Page**

Figure S1	2
Figure S2	2
Figure S3	3
Figure S4	4
Table S1	5
Table S2	16

Supplementary Figure. S1

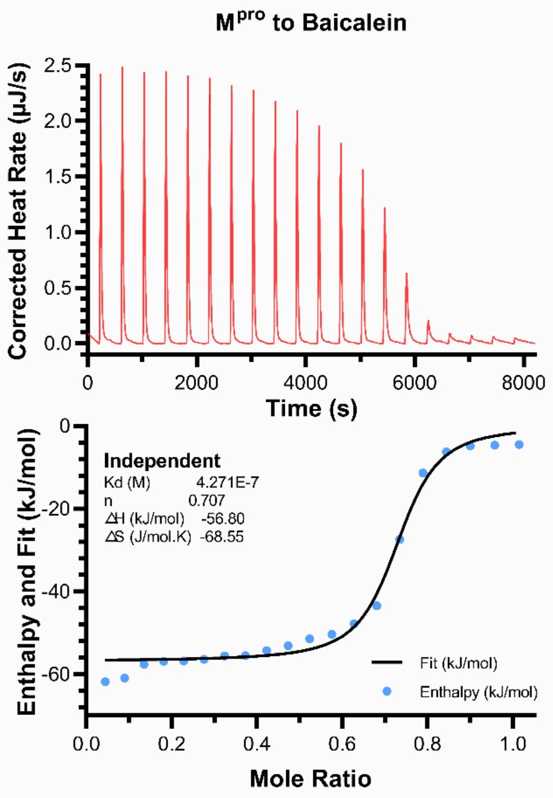

Figure S1. The Isothermal Titration Calorimetry result of M^{pro} to Baicalein.

Supplementary Figure. S2

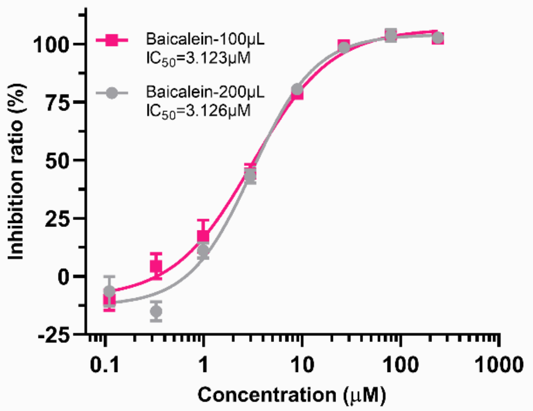

Figure S2. The IC_{50} value and dose-effect relationship curve of baicalein at 100μL/200μL.

Supplementary Figure. S3

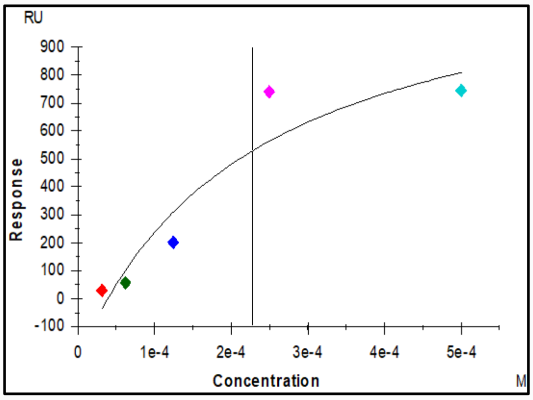

KD (M)	Rmax (RU)	offset (RU)	Chi² (RU²)
2.27E-04			2.59E+04
	1494	-218.3	

Figure S3. Steady analysis fitting curves for Ginkgolic Acid C15:1 to Mpro and related parameters.

Supplementary Figure. S4

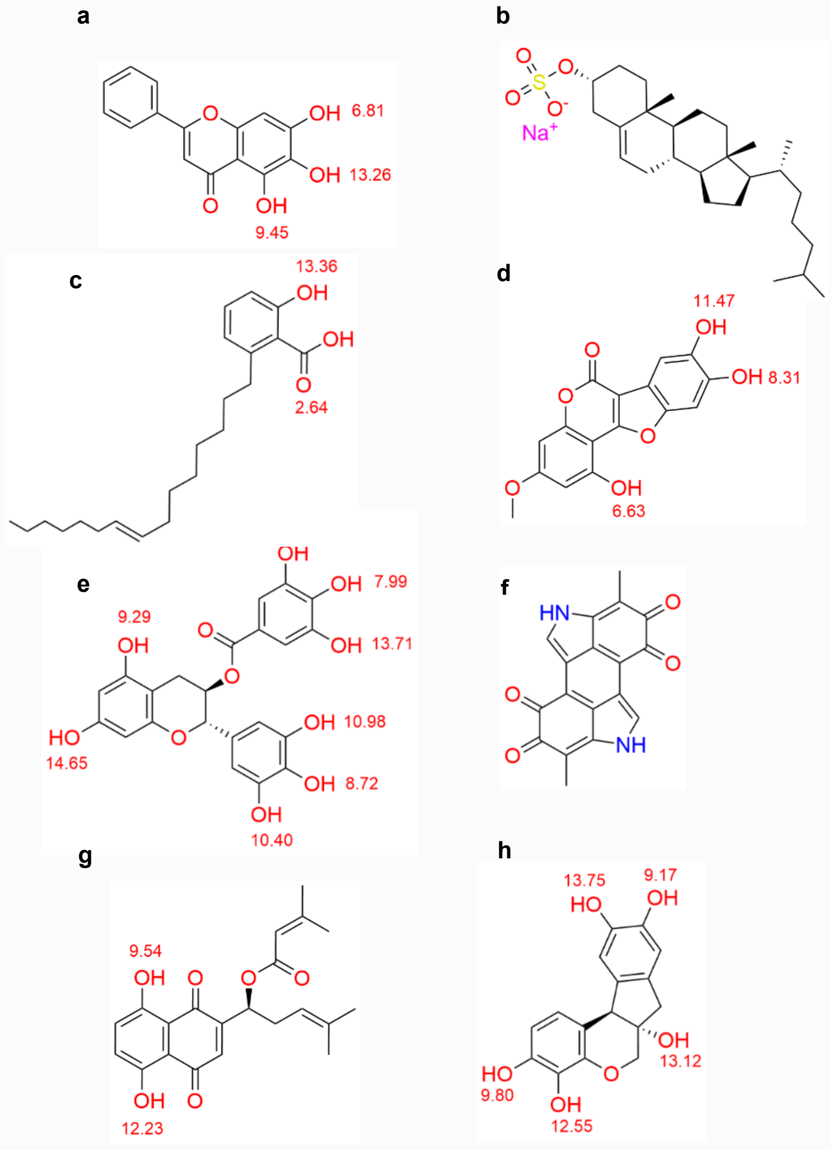

Figure S4. The pKa value of each group of the compound. (a) Baicalein, (b) Cholesteryl sodium sulfate, (c) Ginkgolic Acid C15:1, (d) Wedelolactone, (e) (-)-Gallocatechin Gallate, (f) Melanin, (g) β,β-Dimethylacrylalkannin, (h) Hematoxylin.

Supplementary Table S2.

Serial number	Name	CAS Number	Structure	Inhibition%(80μM)		
	1. Flavonoids					
	(1) flavanones					
1	Corylifol A	775351-88-7	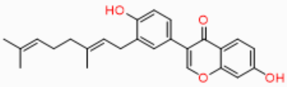	100.00%	100.01%	99.99%
2	Baicalein	491-67-8	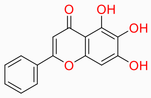	99.94%	100.64%	98.67%
3	3,3',4',5,6,7-Hexahydroxyflavone	90-18-6	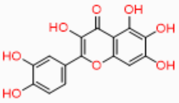	99.40%	98.98%	98.10%
4	Myricetin	529-44-2	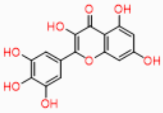	90.64%	90.18%	90.28%
5	Scutellarein	529-53-3	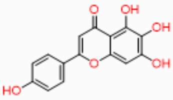	84.13%	85.01%	83.89%
6	Baicalin	21967-41-9	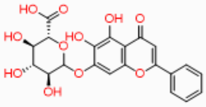	77.46%	72.02%	
7	Breviscapin	116122-36-2	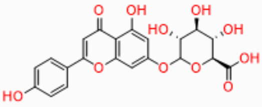	66.01%	78.06%	77.78%

8	Gambogic Acid	2752-65-0	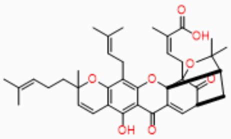	78.46%	78.61%	79.43%
	(2) catechins					
9	(-)-Gallocatechin Gallate	4233-96-9	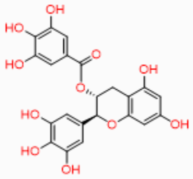	100.69%	102.08%	101.74%
10	(-)-Epigallocatechin Gallate	989-51-5	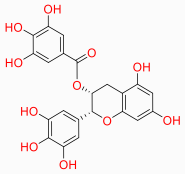	82.60%	85.53%	79.02%
11	(-)-Epigallocatechin	970-74-1	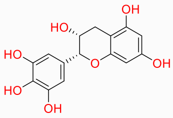	78.95%	75.28%	77.55%
12	Theaflavine-3,3'-Digallate	33377-72-9	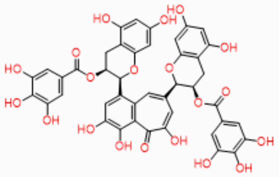	95.00%	94.93%	95.36%
13	Theaflavin 3,3'-Digallate	30462-35-2	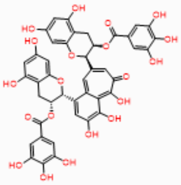	89.63%	87.11%	89.52%

2. Lipids

(1) fatty acids

14	Eicosadienoic Acid	2091-39-6	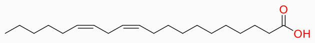	104.73%	103.14%	107.18%
15	Petroselinic Acid	593-39-5	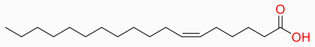	103.91%	105.29%	103.73%
16	Oleic Acid	112-80-1	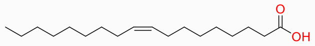	103.98%	104.37%	103.27%
17	Palmitoleic Acid	373-49-9	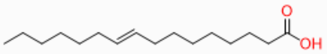	102.92%	103.78%	102.80%
18	10Z-Nonadecenoic acid	73033-09-7	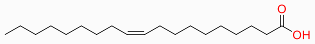	126.52%	101.02%	101.53%
19	Erucic Acid	112-86-7	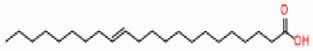	100.63%	99.68%	100.48%
20	Pentadecanoic Acid	1002-84-2	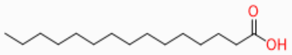	92.31%	92.58%	91.46%
21	Nervonic Acid	506-37-6	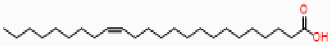	93.60%	89.75%	93.14%
22	Myristic Acid	544-63-8	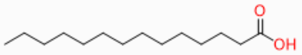	85.01%	82.11%	84.30%
23	Elaidic Acid	112-79-8	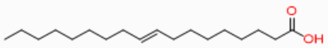	74.89%	79.40%	87.31%
24	Arachidonic Acid	506-32-1	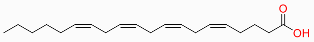	82.20%	76.72%	73.70%

(2) phosphates

25	Phytic Acid	83-86-3	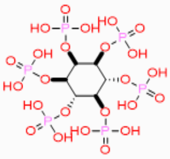	97.60%	97.45%	98.02%
	3. Phenylpropanoids					
26	Wedelolactone	524-12-9	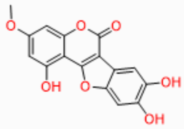	101.95%	101.88%	102.36%
27	Isodemethylwedelolacton	350681-33-3	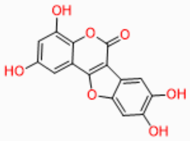	99.67%	99.38%	99.79%
28	Fraxetin	574-84-5	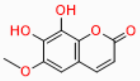	96.84%	97.85%	96.97%
29	4-Methylesculetin	529-84-0	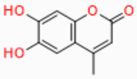	79.12%	79.87%	77.57%
30	Carnosol	5957-80-2	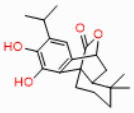	74.48%	73.35%	80.59%
	4. Phenols					
	(1) binary phenols and glycosides					
31	Kukoamine B	164991-67-7	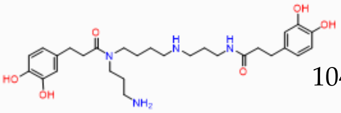	104.18%	103.22%	104.32%

32	1,5-Dicaffeoylquinic Acid	30964-13-7	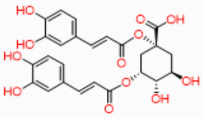	74.57%	75.61%	71.24%
33	Hydroxytyrosol Acetate	69039-02-7	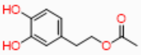	102.98%	103.09%	102.91%
34	4-Methylcatechol	452-86-8	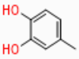	102.69%	102.51%	102.92%
35	3,4-Dihydroxyphenyl ethanol	10597-60-1	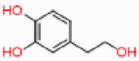	101.17%	102.33%	102.25%
36	Osmundacetone	37079-84-8	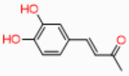	98.66%	98.31%	98.46%
37	Hydrocaffeic Acid	1078-61-1	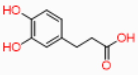	88.27%	90.29%	90.08%
38	3,4-Dihydroxyphenyl acetic Acid	102-32-9	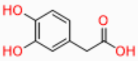	72.40%	71.59%	70.57%
39	Rosmarinic Acid	20283-92-5	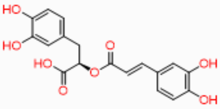	46.31%	76.08%	69.63%
40	9'-Methyl Lithospermate B	1167424-31-8	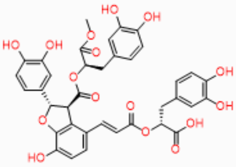	73.57%	72.67%	74.36%
41	Gnetol	86361-55-9	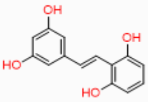	81.51%	81.39%	81.62%
42	Hematoxylin	517-28-2	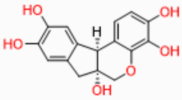	103.72%	103.56%	103.03%

43	Brazilin	474-07-7	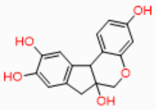	100.86%	101.06%	101.28%
44	(7S)-3,7,10,11-Tetrahydroxy-7,8-dihydro-6H-dibenzo[b,d]oxocin-7-methanol	102036-29-3	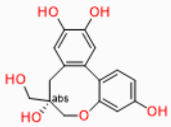	101.15%	101.14%	101.45%
45	Forsythoside E	93675-88-8	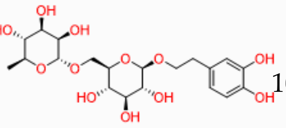	103.35%	102.62%	100.37%
46	Plantainoside D	147331-98-4	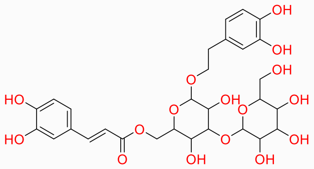	101.38%	99.79%	99.90%
47	Isoforsythiaside	1357910-26-9	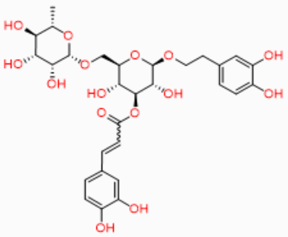	100.42%	100.44%	101.30%
48	Arenarioside	94130-58-2	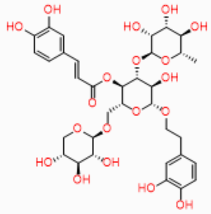	99.11%	99.86%	99.31%
49	Oleuropein	32619-42-4	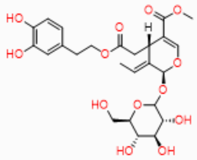	96.62%	97.72%	

50	Plantamajoside	104777-68-6	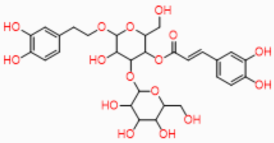	93.60%	93.95%	92.47%
51	Forsythoside B	81525-13-5	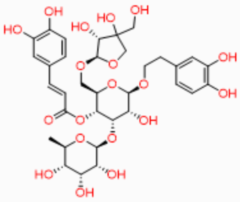	92.91%	95.11%	93.46%
52	Desrhamnosyl Isoacteoside	105471-98-5	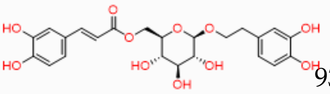	93.35%	94.40%	92.55%
53	Echinacoside	82854-37-3	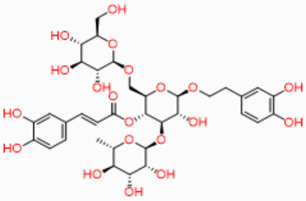	93.62%	90.06%	89.84%
54	2'-Acetylacteoside	94492-24-7	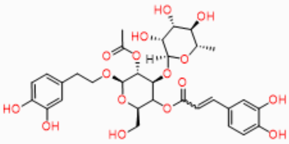	90.56%	93.37%	90.77%
55	Tubuloside A	112516-05-9	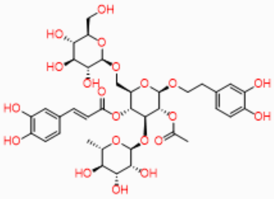	87.62%	90.37%	86.87%
56	Forsythoside A	79916-77-1	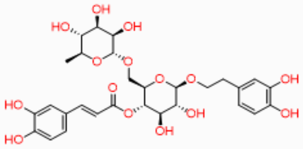	77.01%	73.60%	71.69%
	(2) ternary phenols and glycosides					

57	Propyl Gallate	121-79-9	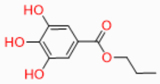	78.46%	78.61%	79.43%
58	Corilagin	23094-69-1	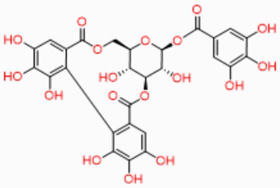	100.82%	101.84%	101.22%
59	Tannic Acid	1401-55-4	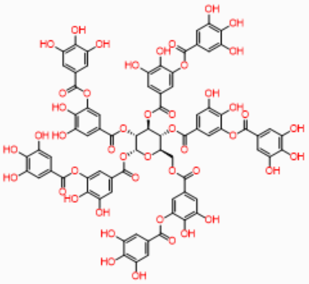	89.58%	88.66%	87.66%
60	Geraniin	60976-49-0	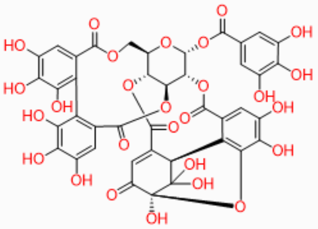	71.62%	70.07%	71.11%
61	Punicalagin	65995-63-3	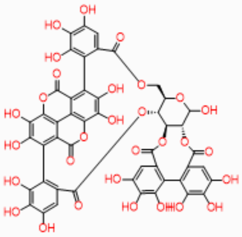	75.03%	73.51%	73.40%

(3) salicylic acids

62	Ginkgolic Acid C15:1	22910-60-7	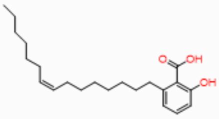	103.73%	107.06%	103.73%

63	Ginkgolic Acid C17:1	111047-30-4	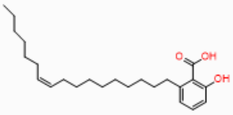	102.36%	100.63%	101.61%
	5. Quinones					
	(1) benzoquinones					
64	2,6-Dimethylbenzoquinone	527-61-7	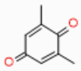	101.69%	101.34%	101.15%
65	Embelin	550-24-3	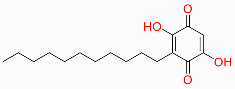	102.98%	89.75%	99.35%
	(2) naphthoquinones					
66	β,β-Dimethylacrylalkannin	34539-65-6	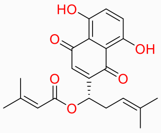	100.45%	99.09%	97.96%
67	3-(Acetyloxy)-3-methylbutanoic acid (1S)-1-(1,4-dihydro-5,8-dihydroxy-1,4-dioxo-2-naphthalenyl)-4-methyl-3-pentenyl ester	69091-17-4	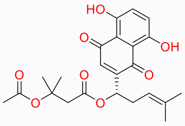	93.12%	92.58%	93.44%
68	(±)-Shikonin	54952-43-1	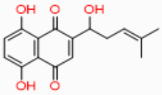	87.60%	95.04%	93.71%
69	Deoxyshikonin	43043-74-9	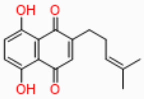	82.31%	88.46%	84.98%

70	(β,β-Dimethylacryl)Shikonin	24502-79-2	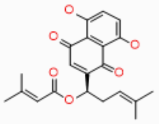	82.39%	82.91%	82.80%
71	5-Hydroxy-1,4-naphthalenedione	481-39-0	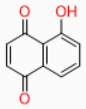	82.51%	81.21%	82.92%
72	β,β-dimethylacrylshikonin	5162-01-6	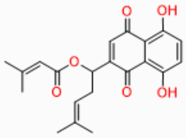	75.31%	77.34%	77.10%
73	Plumbagin	481-42-5	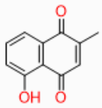	70.20%	74.76%	75.47%
	(3) anthraquinones					
74	Anthraquinone-2-Carboxylic Acid	117-78-2	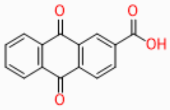	85.44%	84.57%	
	6. Alkaloids					
	(1) isoquinolines					
75	Sanguinarine chloride	5578-73-4	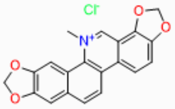	108.35%	108.15%	

76	Sanguinarine	2447-54-3	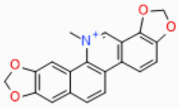	107.86%	107.40%	108.34%
77	Chelerythrine	34316-15-9	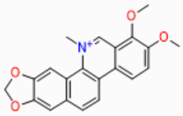	99.38%	98.97%	99.51%
78	Chelerythrine chloride	3895-92-9	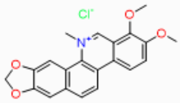	98.66%	98.31%	98.46%
	(2) pyrrolidines					
79	Maleimide	541-59-3	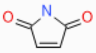	103.98%	104.37%	103.27%
80	Melanin	8049-97-6	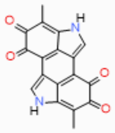	95.85%	96.84%	96.92%
81	Harmol	487-03-6	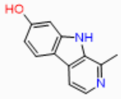	74.50%	81.33%	79.31%
	(3) morpholines					
82	Vanitiolide	17692-71-6	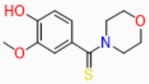	99.14%	99.16%	98.95%
	7. Terpenoids and Steroids					

83	Cholesteryl sodium sulfate	2864-50-8	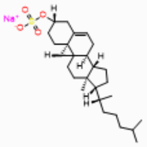	100.46%	100.30%	100.48%
84	Oridonin	28957-04-2	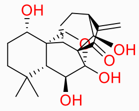	84.28%	83.80%	85.60%
85	Celastrol	34157-83-0	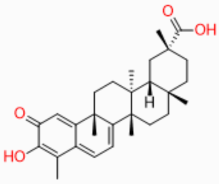	89.61%	85.83%	91.32%
86	Ziyuglycoside II	35286-59-0	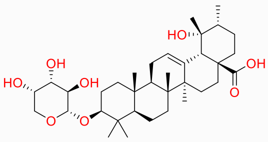	70.21%	74.61%	47.27%

Table S2. 86 compounds obtained from the repeat sieve and their inhibition rates.

Supplementary Table S3.

Capillary ID	Sample ID	Concentration(μM)	Tm(℃)	△T
1	Mpro	/	55.3°C	/
2	Mpro	/	55.3°C	/
3	Mpro+DMSO(1)	0	54.9°C	/
4	Mpro+DMSO(2)	0	54.9°C	/
5	Baicalein(1)	3.125	55.2°C	0.3°C
6	Baicalein(2)	12.5	55.6°C	0.7°C
7	Baicalein(3)	50	56.6°C	1.7°C
8	Baicalein(4)	200	57.0°C	2.1°C
17	Cholesterol sodium sulfate(1)	3.125	55.0°C	0.1°C
18	Cholesterol sodium sulfate(2)	12.5	55.0°C	0.1°C
19	Cholesterol sodium sulfate(3)	50	54.1°C	-0.8°C
20	Cholesterol sodium sulfate(4)	200	48.9°C	-6.0°C
25	Wedelolactone(1)	3.125	55.2°C	0.3°C
26	Wedelolactone(2)	12.5	55.4°C	0.5°C
27	Wedelolactone(3)	50	55.8°C	0.9°C
28	Wedelolactone(4)	200	56.1°C	1.2°C
33	Melanin(1)	3.125	55.0°C	0.1°C
34	Melanin(2)	12.5	54.9°C	0.0°C
35	Melanin(3)	50	54.9°C	0.0°C
36	Melanin(4)	200	54.4°C	-0.5°C
43	β,β-dimethylacrylalkannin(1)	12.5	55.1°C	0.2°C
44	β,β-dimethylacrylalkannin(2)	50	57.7°C	2.8°C
45	β,β-dimethylacrylalkannin(3)	200	58.3°C	3.4°C

Table S3. Tm values for M^{pro} in the presence of different compounds.
